# Effectiveness of Various Final Irrigation Techniques on Sealer Penetration in Curved Roots: A Confocal Laser Scanning Microscopy Study

**DOI:** 10.1155/2020/8060489

**Published:** 2020-04-13

**Authors:** Ayca Yilmaz, Turgut Y. Yalcin, Dilek Helvacioglu-Yigit

**Affiliations:** ^1^Department of Endodontics, Faculty of Dentistry, Istanbul University, Istanbul, Turkey; ^2^Department of Endodontics, Faculty of Dentistry, Kocaeli University, Kocaeli, Turkey

## Abstract

**Objective:**

To compare the efficacy of various techniques used for final irrigation on sealer penetration in the apical one-third of curved root canals. *Material and Methods*. Sixty-five freshly extracted maxillary first molar teeth with mesiobuccal roots having more than 20° of root curvature were used. The root canals were instrumented and randomly divided into four experimental groups and one control group. In the 4 experimental groups, 3 ml of 17% EDTA followed by 3 ml of 5.25% NaOCl was delivered with the use of the following protocols: Group 1: manual dynamic activation (MDA), Group 2: sonic irrigation (SI), Group 3: passive ultrasonic irrigation (PUI), and Group 4: conventional needle irrigation (CI). All teeth were obturated with gutta-percha and AH Plus sealer labeled with fluorescent dye. Transverse sections at 2 mm and 4 mm distance from the root apex were examined with the aid of confocal laser scanning microscopy. Total percentage (%) and maximum depth (*μ*m) of sealer penetration were measured.

**Results:**

All the experimental groups exhibited significantly higher penetration rates than the control group at both sections (*p* < 0.05). However, no significant differences were found in the penetration depth and percentage among the four experimental groups evaluated at both sections (*p* > 0.05).

**Conclusion:**

PUI, SI, and MDA did not significantly improve sealer penetration in the apical portion of curved root canals when compared to conventional needle irrigation.

## 1. Introduction

One of the main objectives of root canal treatment is thorough debridement of the root canal system, eliminating the microorganisms and their metabolic products as well as organic and inorganic substances from the canal space [1]. Chemomechanical preparation of the root canal system is an essential step for endodontic success. The pulp tissue, dentin debris, bacteria, related irritants, and the smear layer should be removed from the root canal system during the procedure of root canal treatment [2, 3]. The smear layer is a 1–2 *μ*m thick amorphous structure created during biomechanical instrumentation [3]. The presence of the smear layer acts as a barrier that may hinder the penetration of root canal irrigants, medicaments, and/or sealers into the dentinal tubules [4]. Although there is some disagreement about whether to remove the smear layer, the general recommendation is that it should be eliminated before obturation [5]. Several reports have noted the effect of smear layer removal on the interaction between the dentin and root filling material [4]. Many irrigant delivery techniques and agitation methods have been proposed to resolve this issue and hence, maintain efficient cleaning and disinfection during final irrigation [6]. Agitation of irrigants may also improve the sealing properties of root canal filling, providing a better seal interface between root filling and canal walls.

Several techniques have been used for root canal irrigation, such as conventional needle irrigation (CI), manual dynamic activation (MDA), sonic irrigation (SI), and passive ultrasonic irrigation (PUI). There are numerous studies which investigate the effect of different agitation techniques on the removal of the smear layer and the sealer penetration into dentinal tubules [7–13]. The mechanical flushing action created by CI is relatively weak [1]. In the MDA technique, it was reported that gently moving a well-fitted gutta-percha master cone up and down in short strokes can produce an effective hydrodynamic effect within the root canal system [14]. It was shown that MDA was significantly more effective than the automated-dynamic irrigation and static irrigation [15].

The EndoActivator System (Dentsply Tulsa Dental Specialties, Tulsa, OK) is a sonically driven canal irrigation system and was reported to be able to effectively clean debris from lateral canals, removing the smear layer within the curved canals [14]. When compared with the conventional needle irrigation technique, it had better results in the removal of the smear layer from the canal walls [16].

EndoUltra (Vista, Racine, Wisconsin) is a cordless ultrasonic device, with a frequency of 40 kHz, inducing acoustic streaming and cavitation [17]. Although PUI was shown to be significantly better than CI, it was reported that PUI with EDTA and NaOCl did not completely remove the smear layer from the apical third of the canal walls [18, 19].

It has been shown that irrigants have a limited effect on smear layer removal closer to the apex, regardless of the irrigation technique [20]. The apical third of the root canal system is especially difficult to clean because of the presence of complex anatomical spaces such as apical deltas, narrow isthmuses, and lateral canals. In the case of curved roots, it is more difficult to achieve complete disinfection in the apical third, where infected dentin and smear layer may remain [21]. Studies on smear layer removal and cleanliness of the curved root canals have shown that activation techniques improve the effectiveness of the final irrigation [21–23]. However, the effectiveness of agitation techniques on sealer penetration has not previously been studied in curved roots. Hence, the aim of the present study was to compare the efficacy of various final irrigation techniques on sealer penetration at distances of 2 mm and 4 mm from the root apex of curved root canals. The tested hypothesis was that the agitation techniques used in this study would be able to improve sealer penetration in the apical portion of curved root canals when compared to conventional needle irrigation.

## 2. Materials and Methods

This study was approved by the Ethical Scientific Committee of Kocaeli (KU GOKAEK 2017/314). Sixty-five maxillary first molar teeth with mesiobuccal roots having more than 20° of root curvature and similar characteristics of length (20-22 mm) were used. Radiographs were taken, and the curvature of mesiobuccal roots was measured, using the technique described by Schneider [24]. Teeth with mesiobuccal roots having canal curvature of less than 20°, immature apices, and previous endodontic treatment were excluded from the study.

Access cavities were prepared. To measure canal length, a #10 K-file was inserted into the canal until visible at the apical foramen. The working length (WL) was established by subtracting 1 mm from this length.

The mesiobuccal canals were prepared using ProTaper Next rotary instruments (Dentsply Maillefer, Ballaigues, Switzerland) to X3 (tip size 30 with the taper of .07). The canals were irrigated with 1 ml of 5.25% sodium hypochlorite (NaOCl) after each file removal. The entire irrigation procedure was performed using 30-gauge irrigation needles (NaviTip, Ultradent Products Inc., South Jordan, UT). A #10 K-file was used to maintain apical patency.

The root canals were randomly assigned to 5 groups according to the final irrigation protocol: one control group (*n* = 5) and four final irrigation groups (*n* = 15). The control group received no further application while in the experimental groups, 3 ml of 17% ethylenediaminetetraacetic acid (EDTA) followed by 3 ml of 5.25% NaOCl was delivered with the use of the following protocols:

1. *Group 1: manual dynamic activation (MDA)*: the canals were flooded with each irrigant, and a well-fitted gutta-percha point (ProTaper Next Gutta-Percha Points X3, Dentsply Maillefer) was gently moved up and down manually at 2 mm short of the WL at an approximate rate of 100 strokes per minute [15]. Each solution was activated for 1 minute, using the pumping master cone method.
2. *Group 2: sonic irrigation (SI)*: each irrigant was activated with the EndoActivator System (Dentsply Maillefer) set at 10,000 cycles per minute (cpm) for 1 minute by using the tip 25/.04 placed within 2 mm of the WL.
3. *Group 3: passive ultrasonic irrigation (PUI)*: an EndoUltra handpiece (Vista, Racine, Wisconsin, USA) with a noncutting NiTi tip 15/.02 was used at a frequency of 40 kHz at 2 mm short of the WL. Each irrigant was passively agitated using the intermittent flush technique, with a total irrigation volume of 3 ml for 3 cycles of 20 seconds. In the intermittent flush technique, the irrigant is injected into the root canal by a syringe and replenished several times after each ultrasonic activation cycle [6].
4. *Group 4: conventional needle irrigation (CI)*: a rinse of 3 ml of 17% EDTA for 1 min and 3 ml of 5.25% NaOCl for 1 min was performed through a syringe needle of 30 gauges. The needle was placed 2 mm short of the working length. The irrigant was dispensed with agitation by moving the needle up and down in the root canal. It is crucial that the needle should remain loose inside the canal during irrigation [6].

After final irrigation, each canal was flushed with 3 ml of distilled water and dried with 3 paper points. All teeth were obturated with a matching tapered gutta-percha cone of the ProTaper Next instruments (ProTaper Next Gutta-Percha Points X3) and AH Plus sealer (Dentsply DeTrey, Konstanz, Germany). The sealer was labeled with 0.1% fluorescent rhodamine B isothiocyanate (Merck 107599 Rhodamine B, Merck Millipore, Darmstadt, Germany). A standard volume of 0.05 ml of sealer was delivered into the root canal. A #25 lentulo spiral was used with a low-speed handpiece at a speed of 300 rpm with an up-and-down motion within the canal six times.

With the use of a hot instrument, excess gutta-percha in the access cavity was removed and temporary filling material (Coltosol, Coltene-Whaledent, Altstatten, Switzerland) was placed. All procedures were performed by the same operator. Teeth were incubated at 37°C and 100% humidity for 14 days to allow the root canal sealer to set. Transverse sections were obtained using a water-cooled 0.3 mm microtome saw (IsoMet 1000 Precision Cutter, Buehler, Illinois, USA) at 2 mm and 4 mm distance from the root apex to obtain sections of 200 *μ*m thickness. The coronal surfaces of the slices were polished with silicon abrasive carbide paper to remove dentin debris created during the sectioning procedures. The apical surfaces of the samples were mounted on glass slides, which were numbered. All sections were then examined using confocal laser scanning microscopy (CLSM) (Leica TCS SPE, Leica Microsystems, Wetzlar, Germany) with the aid of a solid laser (532 nm). A dry lens (numeric aperture 0.3) at ×10 magnification was used to observe the samples. Images were acquired and analyzed using Leica Application Suite Advanced Fluorescence 3.3 software (Leica Microsystems).

The percentage of the sealer penetration was determined by measuring the regions where sealer penetrated into the dentinal tubules along the root canal walls using the ImageJ measurement tool (https://imagej.nih.gov/ij/), and then this value was divided by the circumference of the root canal wall, and this result was multiplied by 100 to calculate the percentage.

Wherever the maximum depth of sealer penetration along the root canal circumference could not be evaluated in a single image, additional partial images were taken and processed using CorelDRAW Graphics Suite X5 (Corel Corporation, Ottawa, Canada). These were then imported to the ImageJ program to measure maximum depth (*μ*m) of sealer penetration.

Statistical analyses were performed using Number Cruncher Statistical System 2007 (NCSS, Kaysville, Utah, USA), and significance was set at the 5% level (*p* < 0.05). The Shapiro–Wilk test was used to verify the assumption of normality. The nonparametric Kruskal-Wallis test, followed by Dunn post hoc tests, was used to compare sealer penetration in each group. Sealer penetration at 2 mm and 4 mm levels in each group was analyzed using the Wilcoxon signed rank sum test.

## 3. Results

**Figure 1:**
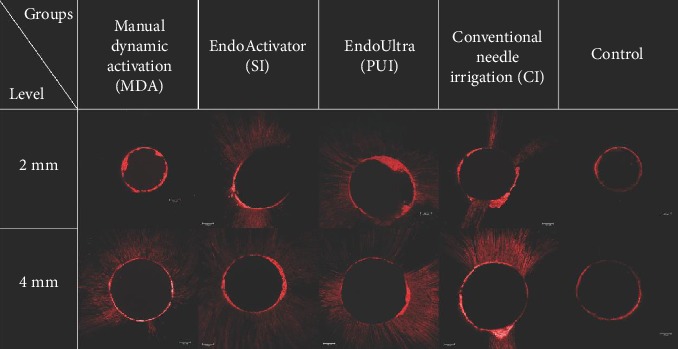
The confocal laser scanning microscopic images from selected samples at the 2 and 4 mm sections representing different irrigation methods.

A representative CLSM image of a sample from each group at the 2 mm and 4 mm levels is shown in Figure 1.  Table 1 shows the percentage of sealer penetration values (mean ± SD; median (IQR)) among all groups at the 2 mm and 4 mm sections. All experimental groups exhibited a significantly higher percentage of the sealer penetration than the control group at both sections (*p* < 0.05). However, no significant differences were found among the four experimental groups at both sections (*p* > 0.05). While the PUI, SI, and CI groups showed significantly less percentage penetration in 2 mm sections as compared to the 4 mm section (*p* < 0.05), there was no significant difference between sections in the MDA group (*p* > 0.05).

The maximum depth of sealer penetration (*μm*) values (mean ± SD; median (IQR)) in both sections is presented in Table 2. No significant differences in penetration depth were found among the four experimental groups evaluated at both sections (*p* > 0.05). The control group demonstrated significantly lower values than all experimental groups (*p* < 0.05). The PUI and SI groups showed significantly less depth of penetration in 2 mm sections as compared to 4 mm sections (*p* < 0.05).

## 4. Discussion

It has widely been considered that the smear layer serves as a barrier blocking the penetration of root canal irrigants, medicaments, and root canal sealers into the dentinal tubules [3]. Despite its benefits, removal of the smear layer is still contentious in the literature [3, 25]. Less coronal leakage as a result of smear layer removal has been reported [26]. One possible explanation is the effect of smear layer removal on sealer penetration into the dentinal tubules [27]. Consequently, CLSM evaluation of the amount and depth of sealer penetration is commonly used to investigate the effect of irrigant agitation on the cleanliness of root canal walls. However, sealer penetration studies assessing the efficiency of agitation methods have tended to focus on straight canals, and to the best of our knowledge, there is no article in the literature regarding sealer penetration efficiency of activation techniques in curved root canals. In the present study, the efficacy of different techniques used for final irrigation on sealer penetration of curved root canals was compared. The results indicate that the tested hypothesis was rejected because there was no difference in sealer penetration, regardless of the irrigation techniques used.

Delivery and replenishment of irrigants at the apical portions of the root is a challenging aspect of apical irrigation [28]. The apical root dentin exhibits few or no tubular characteristics [29], and recent evidence suggests that smear layer removal is less effective in that portion of the root than in the coronal parts [30–32]. This finding prompted research investigating the smear layer removal ability of irrigants and irrigation protocols, specifically from the apical third of the root canal. The present study assessed the efficiency of irrigation techniques at the apical sections obtained at lengths of 2 mm and 4 mm when measured from the apex. Unlike other studies in which the teeth were decoronated [8, 9], the crown portions of the teeth were not removed in the present study prior to root canal preparation. However, the access cavities having four walls provided a reservoir for irrigants to be continuously refreshed and exchanged during activation.

Conventional needle irrigation is a widely accepted technique that uses needles of variable gauges, either passively or with agitation. The depth of needle penetration and the volume of irrigant can be easily controlled in this technique whereas fluid flow rate during irrigation is difficult to control and standardize [6]. With the recent introduction of new irrigation modalities, activation systems have become one of the most essential components of chemomechanical debridement of the root canal system. In this study, among the four different irrigation techniques, no significant differences were observed in the investigated parameters in relation to sealer penetration. While these findings align well with some of the earlier studies [7, 9], the results of other studies in a review of the literature were found to be contrasting [10, 11, 13, 33–35]. Galler et al. [34] found greater penetration depths in the apical thirds for ultrasonic and sonic activation groups compared to MDA. Barbosa et al. [35] reported that the ultrasonic activation provides better penetration of the sealer than MDA did. Machado et al. [13] also concluded that SI achieved better degrees of tubular dentin sealer penetration, compared with the CI. The main factor affecting these contrasting findings could be the sample selection. All these studies were carried out on straight root canals whereas curved root canals were used in the present study which is a challenging issue to deliver the irrigation solution to the apical part regardless of the irrigation technique. In particular, the irrigant used, the selected concentration and volume of the irrigation, and the technique and duration of agitation of the irrigant may influence the results of studies on the effectiveness of agitation methods. These conflicting results may also be attributable to the different criteria and methods of evaluation used in various studies [6].

Sealer penetration into the dentinal tubules is considered a desirable outcome of root canal filling, as it improves sealing ability and mechanical retention of the filling [30]. Moreover, sealers within dentinal tubules might trap residual bacteria in tubules and inhibit bacterial colonization [36]. Theoretically at least, broader sealer penetration along the circumference of the root canal is thought to achieve a better seal. According to our results, the percentage of penetration was significantly higher in the experimental groups than in the control group, indicating irrigation with activation systems as well as activation with a syringe/needle to be effective in smear layer removal. Final irrigation and activation of irrigants yielded higher sealer penetration into dentinal tubules when compared to no final irrigation.

Complete disinfection of the apical part of curved root canals presents a major challenge. Irrigant activation offers no known benefit in relation to debris and smear layer removal in the apical portions of curved root canals [12, 21]. This result has been attributed to the severity of instrument contact with curved root canal walls, resulting in restricted oscillation and dampening the effect of the instrument within the canal [37]. Unlike other research in this area, the present study shows that PUI and sonic irrigation are as effective as conventional needle irrigation. One possible explanation for this unexpected finding is that the level of needle penetration was similar for all irrigation techniques. Another possible explanation relates to the limitations imposed by curved root canals; although PUI has proved effective for debris and smear layer removal in straight canals [16], this device has some limitations, especially in curved canals. As there is a risk of touching the canal walls and causing new smear layer formation [22], it is reasonable to hypothesize that this limits the potential benefits of PUI.

In the present study, the percentage of the sealer penetration in the 4 mm sections was greater than that in the 2 mm sections for all except the MDA group. The difference between apical and coronal sections has also been noted in previous studies [5, 7, 38]. One possible explanation for this difference is the decreased density and diameter of dentinal tubules in the apical root dentin, with some areas completely devoid of tubules [29, 39]. Additionally, it is more difficult to remove the smear layer from the apical third of the root canal than the middle third because of reduced irrigant delivery [40].

## 5. Conclusions

In conclusion, irrigation using PUI, SI, and MDA did not significantly improve sealer penetration when compared to conventional needle irrigation in curved root canals. Further investigation of other irrigation modalities and systems may yield additional insights into how these methods can improve sealer penetration in challenging systems such as curved roots.

## Figures and Tables

**Table 1:** Percentage of sealer penetration (mean ± SD; median (IQR)) at the 2 and 4 mm sections.

Percentage of penetration (%)		2 mm	4 mm	*p* ^∗^
MDA	Mean ± SD	35.31 ± 29.87^a^	44.89 ± 26.93^a^	0.372
	Median (IQR)	32.87 (11.73-54.61)	44.67 (30.76-47.89)	

SI	Mean ± SD	39.6 ± 21.62^a^	52.68 ± 20.96^a^	0.130
	Median (IQR)	42.32 (28.27-52.97)	51.27 (39.61-67.82)	

PUI	Mean ± SD	35.26 ± 28.95^a^	55.96 ± 25.37^a^	0.065
	Median (IQR)	33.1 (11.21-55.7)	52.67 (32.01-63.52)	

CI	Mean ± SD	35.93 ± 23.03^a^	49.27 ± 18.28^a^	0.101
	Median (IQR)	32.82 (13.71-54.66)	51.27 (37.49-65.32)	

Control	Mean ± SD	0.87 ± 1.2^b^	1.56 ± 1.5^b^	0.373
	Median (IQR)	0 (0-2.18)	2 (0-2.91)	

	*p* ^∗∗^	0.043	0.003	

MDA: manual dynamic activation; SI: sonic irrigation; PUI: passive ultrasonic irrigation; CI: conventional needle irrigation; SD: standard deviation; IQR: the interquartile range. ^∗^Mann-Whitney *U* test (*p* < 0.05). ^∗∗^Kruskal-Wallis test (*p* < 0.05). Superscript letters show statistical difference in a column. Dunn's multiple comparison test (*p* < 0.05).

**Table 2:** Maximum depth of sealer penetration (*μ*m) (mean ± SD; median (IQR)) at the 2 and 4 mm sections.

Maximum depth of sealer penetration (*μ*m)		2 mm	4 mm	*p* ^∗^
MDA	Mean ± SD	430.9 ± 420.57^a^	652.69 ± 385.26^a^	0.169
	Median (IQR)	450.59 (0-729.29)	601.07 (399.1-1095.35)	

SI	Mean ± SD	302.61 ± 324.98^a^	504.67 ± 455.42^a^	0.210
	Median (IQR)	272.15 (0-633.08)	338.4 (0-842)	

PUI	Mean ± SD	378.95 ± 288.87^a^	568.95 ± 269.8^a^	0.065
	Median (IQR)	247.45 (157.43-656.83)	579.99 (335.1-823)	

CI	Mean ± SD	470.06 ± 355.28^a^	5369.17 ± 18594.1^a^	0.467
	Median (IQR)	511.11 (64.53-725.68)	524.05 (307-900.55)	

Control	Mean ± SD	24.55 ± 33.73^b^	38.97 ± 36.5^b^	0.577
	Median (IQR)	0 (0-61.37)	53.32 (0-70.77)	

	*p* ^∗∗^	0.036	0.048	

MDA: manual dynamic activation; SI: sonic irrigation; PUI: passive ultrasonic irrigation; CI: conventional needle irrigation; SD: standard deviation; IQR: the interquartile range. ^∗^Mann-Whitney *U* test (*p* < 0.05). ^∗∗^Kruskal-Wallis test (*p* < 0.05). Superscript letters show statistical difference in a column. Dunn's multiple comparison test (*p* < 0.05).

## Data Availability

The data used to support the findings of this study are available from the corresponding author upon request.
